# Supplementary material for: Ab initio machine-learning simulation of calcium carbonate from aqueous solutions to the solid state
Source: Proc Natl Acad Sci U S A. 2025 Oct 6;122(41):e2415663122. doi: 10.1073/pnas.2415663122 (PMC12541341; doi:10.1073/pnas.2415663122)
Supplement: Supplementary file 1 — Appendix 01 (PDF) [file pnas.2415663122.sapp.pdf]

PNAS

Supporting Information for

Ab initio machine learning simulation of calcium carbonate from aqueous solutions to the solid state

Pablo M. Piaggi, Julian D. Gale and Paolo Raiteri

Pablo M. Piaggi.
E-mail: pm.piaggi@nanogune.eu

This PDF file includes:

Supporting text
Fig. S1

Supporting Information Text

Free energy curves for ion pairing. In Figure S1, we show the free energy curves for ion pairing computed using the SCAN-ML model, enhanced-sampling calculations, and three different system sizes. As described in the Materials and Methods section in the main part of our work, we used systems with one ion pair (Ca^{2+} and ${CO_3}^{2-}$) and 197, 1576, and 5319 water molecules in cubic boxes with side lengths of approximately 18, 36, and 53 Å, respectively. We also computed the error for each system size using four independent simulations, each 20-ns long. The results show that the free energy $G(r)$ as a function of the distance between Ca and C atoms agree with each other within the statistical uncertainty in their corresponding region of validity, i.e., $r < L/2$ where L is the box side length. The difference between free energy curves for different system sizes is around 1 kJ/mol, which is smaller than the thermal energy at room temperature, namely, $k_B T \approx 2.5$ kJ/mol with $T = 300$ K and k_B the Boltzmann constant. This test ensures that the free energies reported in the main part are not influenced by artificial interaction with periodic images, which can happen for small cells as often used for AIMD. Although the free energy curves do not depend on the system size, simulating the larger system sizes allows us to access the free energies at larger distances r,

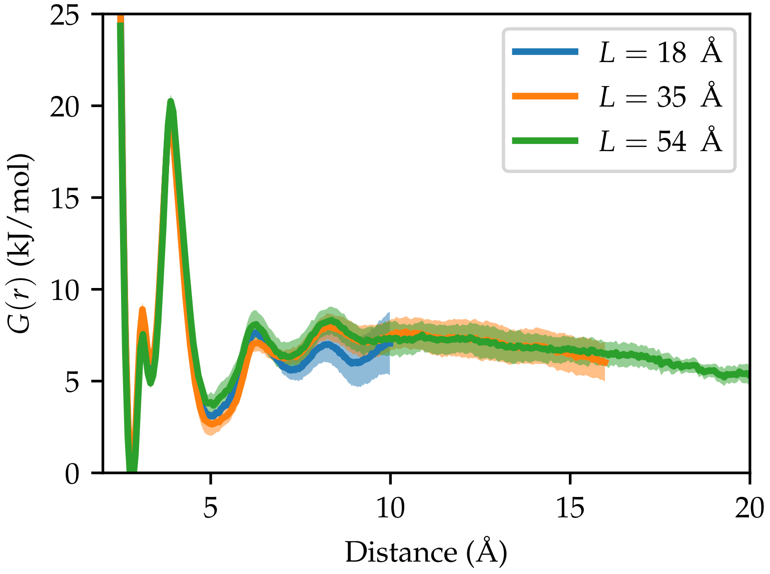

Fig. S1. Free energy $G(r)$ as a function of the distance r between Ca and C atoms for three different system sizes labeled by the box side length. The shaded area represents the standard deviation of the mean computed from four independent simulations. The results show that the free energy curves agree with each other within the statistical uncertainty and thus do not depend on the system size.
